# Effectiveness of ChAdOx1 nCoV-19 (Vaxzevria) primary series vaccine against SARS-CoV-2 beta and delta variants: a nationwide study

**DOI:** 10.1186/s12879-025-11410-7

**Published:** 2025-08-17

**Authors:** Hiam Chemaitelly, Houssein H. Ayoub, Peter Coyle, Patrick Tang, Mohammad R. Hasan, Hadi M. Yassine, Asmaa A. Al Thani, Zaina Al-Kanaani, Einas Al-Kuwari, Andrew Jeremijenko, Anvar Hassan Kaleeckal, Ali Nizar Latif, Riyazuddin Mohammad Shaik, Hanan F. Abdul-Rahim, Gheyath K. Nasrallah, Mohamed Ghaith Al-Kuwari, Adeel A. Butt, Hamad Eid Al-Romaihi, Mohamed H. Al-Thani, Abdullatif Al-Khal, Roberto Bertollini, Laith J. Abu-Raddad

**Affiliations:** 1 Infectious Disease Epidemiology Group, Weill Cornell Medicine-Qatar, Qatar Foundation - Education City, Cornell University, P.O. Box 24144, Doha, Qatar; 2 Department of Population Health Sciences, Weill Cornell Medicine, Cornell University, New York, NY USA; 3 Mathematics Program, Department of Mathematics, Statistics and Physics, College of Arts and Sciences, Qatar University, Doha, Qatar; 4 Hamad Medical Corporation, Doha, Qatar; 5 Department of Biomedical Science, College of Health Sciences, QU Health, Qatar University, Doha, Qatar; 6 Wellcome-Wolfson Institute for Experimental Medicine, Queens University, Belfast, UK; 7 Department of Pathology and Laboratory Medicine, University of British Columbia, Vancouver, Canada; 8 Department of Pathology and Molecular Medicine, McMaster University, Hamilton, Canada; 9 Biomedical Research Center, QU Health, Qatar University, Doha, Qatar; 10 Department of Public Health, College of Health Sciences, QU Health, Qatar University, Doha, Qatar; 11 Primary Health Care Corporation, Doha, Qatar; 12 College of Medicine, Qatar University, Doha, Qatar; 13 Department of Medicine, Weill Cornell Medicine, Cornell University, New York, NY USA; 14 Ministry of Public Health, Doha, Qatar; 15 College of Health and Life Sciences, Hamad bin Khalifa University, Doha, Qatar

**Keywords:** COVID-19, Vaccine, Effectiveness, Vaxzevria, Astrazeneca, ChAdOx1 nCoV-19, Case-control

## Abstract

**Background:**

This study assessed the real-world effectiveness of the ChAdOx1 nCoV-19 vaccine in adults against severe acute respiratory syndrome coronavirus 2 (SARS-CoV-2) infection, symptomatic infection, and severe coronavirus disease 2019 (COVID-19) during periods of Beta and Delta variant dominance in Qatar.

**Methods:**

A national, matched, test-negative case-control study was conducted using 186,130 PCR-positive tests (cases) and 667,289 PCR-negative tests (controls) collected between January 1 and December 18, 2021. Subgroup analyses were performed to evaluate vaccine effectiveness across key strata.

**Results:**

The median time between the first and second doses was 61 days (interquartile range, 56–64 days). Two-dose primary-series effectiveness was 66.0% (95% CI, 55.1–74.3%) against any SARS-CoV-2 infection and 73.0% (95% CI, 44.1–87.0%) against symptomatic infection. Effectiveness was estimated at 100% (95% CI, 64.0–100%) against any Beta variant infection and 65.3% (95% CI, 54.2–73.8%) against any Delta infection. Protection against any infection of any variant peaked at 78.4% (95% CI, 50.7–90.5%) within the first month after the second dose, gradually declining to 45.6% (95% CI, 5.5–68.7%) after 150 days. Effectiveness against severe, critical, or fatal COVID-19, irrespective of variant, was 100% (95% CI, 49.3–100%), with no vaccinated individuals progressing to severe, critical, or fatal disease after infection. Effectiveness of a single dose was 59.9% (95% CI, 51.0–67.3%) against any infection—65.0% (95% CI, 49.7–75.6%) against Beta and 55.9% (95% CI, 43.8–65.5%) against Delta—78.4% (95% CI, 60.9–88.0%) against symptomatic infection, and 100% (95% CI, 88.9–100%) against severe, critical, or fatal COVID-19.

**Conclusion:**

The ChAdOx1 nCoV-19 vaccine provided substantial protection against infection and strong protection against severe outcomes during periods dominated by the Beta and Delta variants, although protection against infection waned within the first few months following the primary series.

**Supplementary Information:**

The online version contains supplementary material available at 10.1186/s12879-025-11410-7.

## Introduction

Multiple vaccines were developed to reduce the acquisition and severity of severe acute respiratory syndrome coronavirus 2 (SARS-CoV-2) infection [1]. While mRNA-based vaccines demonstrated high efficacy [2, 3], their high cost and dependence on cold-chain storage posed logistical challenges, particularly in resource-limited settings [4]. In contrast, the ChAdOx1 nCoV-19 vaccine (AZD1222 or Vaxzevria), an affordable viral vector-based platform, is stable at standard refrigeration temperatures (2–8 °C) for at least six months [5], greatly enhancing its feasibility for vaccination scale-up in regions with inadequate healthcare infrastructure [4].

Randomized controlled trials (RCTs) have demonstrated considerable protection for the ChAdOx1 nCoV-19 primary series against SARS-CoV-2 infection [6–8]. Efficacy against symptomatic infection with the Wuhan virus, more than 14 days after the second dose, was 62.1% in an RCT conducted in the United Kingdom (UK) and Brazil [6], and 74% in a separate RCT conducted across the United States, Chile, and Peru [7]. Further analysis from the UK trial found an efficacy of 66.7% against symptomatic infection with the Alpha variant [8].

However, an RCT in South Africa suggested no efficacy against infection with the Beta variant, raising concerns over the vaccine’s utility, though the results carried substantial statistical uncertainty [9]. Subsequent final analysis of this trial estimated the vaccine’s efficacy at 90.6% against infection with the Wuhan virus, 77.1% against infection with the Delta variant, and only 6.7% against infection with the Beta variant but also with considerable statistical uncertainty [10].

Qatar launched its COVID-19 immunization program in late December 2020 using mRNA vaccines [11]. The ChAdOx1 nCoV-19 vaccine was subsequently introduced for a brief period to accelerate vaccination efforts and was administered through a few mass public vaccination campaigns. However, its use in these campaigns was rapidly discontinued following the negative findings of the South African trial against the Beta variant [9], which was also circulating in Qatar at the time [12], along with reports of rare blood clotting events in other countries [13, 14].

Although the ChAdOx1 nCoV-19 vaccine is no longer in use worldwide, particularly following the emergence of the Omicron variant and the availability of updated vaccines better matched to circulating strains, this study was conducted to complete the evidence base on the real-world effectiveness of first-generation vaccines. It offers insights from one of the most comprehensively studied national populations for COVID-19 vaccine effectiveness [15–22] and may help guide the development of future viral vector vaccines.

This study investigates the effectiveness of the two-dose primary series of the ChAdOx1 nCoV-19 vaccine against SARS-CoV-2 infection, symptomatic infection, and severe (acute-care hospitalization) [23], critical (intensive-care-unit hospitalization) [23], and fatal [24] coronavirus disease 2019 (COVID-19) during periods dominated by the Beta and Delta variants. Vaccine effectiveness against infection was assessed overall and stratified by variant type, prior infection status, clinical vulnerability status, and time since the second dose. In addition, the study includes an analysis of the effectiveness of a single vaccine dose against any infection—both overall and stratified by variant—as well as against symptomatic infection and severe, critical, or fatal COVID-19.

## Methods

This study builds on previous research assessing COVID-19 vaccine effectiveness in Qatar using a test-negative, case-control design [15–17, 25, 26]. Comprehensive descriptions of this methodological approach have been published elsewhere [15–17, 25, 26]. Key methods are briefly summarized below.

### Study population and data sources

This nationwide study was conducted in Qatar from January 1, 2021 to December 18, 2021, covering the period between the launch of the COVID-19 primary-series vaccination campaign [15, 16] and the introduction of the Omicron variant on December 19, 2021 [27]. Early in the year, infection incidence was driven by the Alpha [28] and subsequently the Beta [12] variants, before transitioning into an extended phase dominated by the Delta variant [25] (Supplementary Fig. 1).

Data were sourced from the integrated national digital-health platform (Supplementary Sect. 1), which includes comprehensive information on COVID-19 testing, vaccination, hospitalizations, and deaths. Since the pandemic onset, all SARS-CoV-2 polymerase chain reaction (PCR) tests, regardless of facility or location, have been captured with no missing data (Supplementary Sect. 2). Approximately 5% of Qatar’s population underwent weekly testing during the study period, primarily for routine screening, leading to most infections being identified preemptively rather than through symptomatic testing [17, 18].

Demographic details (sex, age, nationality) were obtained from the national health registry; notably, around 89% of Qatar’s diverse population are expatriates from over 150 countries [29]. COVID-19 vaccines were administered free of charge to all residents and were closely tracked nationwide [11, 17]. More extensive descriptions of the population and data sources have been documented elsewhere [17–20, 29–32].

The ChAdOx1 nCoV-19 vaccine allows flexibility in the interval between the first and second doses, typically ranging from 4 to 12 weeks [6, 33, 34]. Although initial trials used a 4-week interval, subsequent analyses explored extended dosing schedules [6, 33]. Based on these findings, the World Health Organization (WHO) recommended an interval of 8–12 weeks [34, 35]. Many countries adopted this recommendation—or even longer intervals—driven by logistical considerations and emerging immunological evidence [34, 36, 37]. In Qatar, an 8-week interval was adopted as the target schedule for the primary series, reflecting both the logistical demands of mass, field-based vaccination campaigns and the evolving evidence on the vaccine’s immunogenicity at the time.

### Study design

The ChAdOx1 nCoV-19 primary-series vaccine effectiveness against SARS-CoV-2 infection was estimated using a test-negative, case-control design [16, 17, 25, 38–40]. Cases were defined as PCR-positive tests and controls as PCR-negative tests, both conducted during the study period. Effectiveness was determined by comparing the odds of having received the two-dose primary series between cases and controls.

Cases or controls with a PCR-positive result in the preceding 90 days were excluded to avoid misclassification of prolonged positivity as a prior infection [41–43], while those with earlier infections (≥ 90 days) were classified as having a prior infection. Cases or controls who had received any mRNA vaccine dose, or a single dose or more than two doses of the ChAdOx1 nCoV-19 vaccine, were also excluded. Tests conducted within 14 days of the second vaccine dose were additionally excluded.

Cases and controls were matched exactly one-to-five by sex, 10-year age group, nationality, number of coexisting conditions (0 to ≥ 6; Supplementary Sect. 3), prior infection status, PCR test calendar week, and reason for testing, with matching ratio chosen to enhance statistical precision. This matching approach minimized confounding based on known risk factors for infection risk [29, 44–47], as informed by findings of earlier studies on Qatar’s population [11, 16, 17, 48–50].

Effectiveness was estimated both against any SARS-CoV-2 infection, as well as against symptomatic infection by restricting the analysis to tests conducted for clinical suspicion of respiratory symptoms. Variant-specific effectiveness was further assessed by restricting the analysis to periods dominated by each variant: March 8–May 31, 2021, for Beta [51, 52], and June 1–December 18, 2021, for Delta [25, 51, 52]. This time-based classification of circulating variants was informed by viral genome sequencing and variant genotyping conducted throughout the pandemic, as detailed in previous publications [16, 17, 19, 25, 53–55] and Supplementary Sect. 2.

To assess protection against severe outcomes, cases that progressed to severe [23], critical [23], or fatal [24] COVID-19 were matched exactly one-to-five to controls. Severity was determined by trained medical personnel based on independent chart reviews, following the WHO guidelines (Supplementary Sect. 4). All hospitalized patients with PCR-confirmed infections were assessed for disease severity every three days until discharge or death. In this study, individuals were classified based on their worst outcome starting with death, followed by critical, then severe illness.

### One-dose vaccine effectiveness against infection and severe COVID-19

An additional analysis evaluated the effectiveness of a single dose of the ChAdOx1 nCoV-19 vaccine, starting 14 days after administration, against any SARS-CoV-2 infection—both overall and stratified by variant—as well as against symptomatic infection and severe, critical, or fatal COVID-19, using the same methodological approach as for the two-dose primary series.

### Statistical analysis

All PCR test records were reviewed for case and control selection, but only matched samples were included in the analysis. Cases and controls were described using frequency distributions and measures of central tendency, with standardized mean differences (SMDs) ≤ 0.1 indicating adequate balance [56].

Odds ratios (ORs), comparing the odds of primary series vaccination between cases and controls, and associated 95% confidence intervals (CIs), were estimated using conditional logistic regression. CIs were not adjusted for multiple comparisons. Interactions were not examined. Unvaccinated individuals served as the reference group in all estimations.

Vaccine effectiveness and corresponding 95% CI were calculated as 1-OR if the OR was < 1, and as 1/OR-1 if the OR was ≥ 1 [20, 57]. This approach yields a symmetric scale from − 100 to 100% for intuitive interpretation of both positive and negative effectiveness [20, 57]. When the conditional logistic regression failed to converge because of zero vaccinated cases, the 95% CI was calculated using McNemar’s test, using the total number of matched pairs in the 1:n design, as done in earlier studies [26, 51].

Additional analyses were conducted to estimate vaccine effectiveness by time since the second dose (first month and subsequently by 2-month intervals), prior infection status, age (< 50 years; ≥50 years), and clinical vulnerability status. Individuals < 50 years with ≤ 1 comorbidity were considered less clinically vulnerable to severe COVID-19, while those ≥ 50 years or < 50 years but with ≥ 2 comorbidities were classified as more vulnerable to severe COVID-19 [20, 30].

Statistical analyses were conducted in STATA/SE version 18.0 (Stata Corporation, College Station, TX, USA).

### Ethics approval, patient consent, and oversight

The study was approved by the institutional review boards at Hamad Medical Corporation (reference number: MRC–01–20–1078) and Weill Cornell Medicine–Qatar (reference number: 20–00017) and a waiver of informed consent was also approved by the institutional review boards at Hamad Medical Corporation (reference number: MRC–01–20–1078) and Weill Cornell Medicine–Qatar. This study was conducted in accordance with the ethical principles outlined in the Declaration of Helsinki. The study was reported following the Strengthening the Reporting of Observational Studies in Epidemiology (STROBE) guidelines (Supplementary Table 1).

## Results

### Study population

Between January 1, 2021 and December 18, 2021, the study’s end date, 33,457 individuals received two or more doses of the ChAdOx1 nCoV-19 vaccine. For comparison, during the same period, 2,175,216 individuals received two or more doses of an mRNA COVID-19 vaccine.

**Fig. 1:**
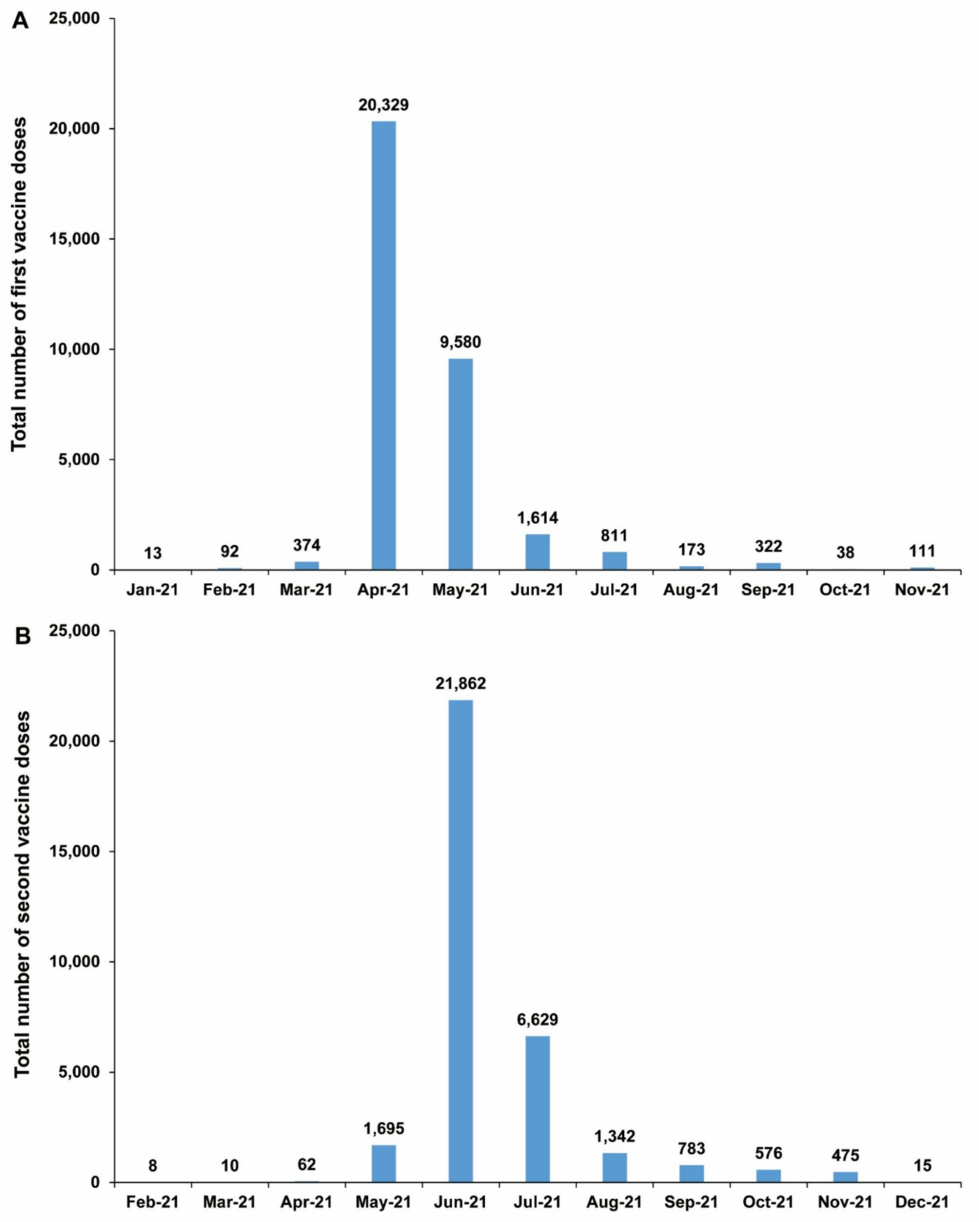
Distribution of A first and B second doses of the ChAdOx1 nCoV-19 (Vaxzevria) vaccine during the study period

The median date for the first ChAdOx1 nCoV-19 dose was April 28, 2021 (interquartile range (IQR), April 26, 2021–May 20, 2021). The median date for the second dose was June 29, 2021 (IQR, June 28, 2021–July 18, 2021). The median time between the first and second vaccine doses was 61 days (IQR, 56–64 days). Figure 1 presents the distribution of first and second doses of the ChAdOx1 nCoV-19 vaccine during the study period.

**Fig. 2:**
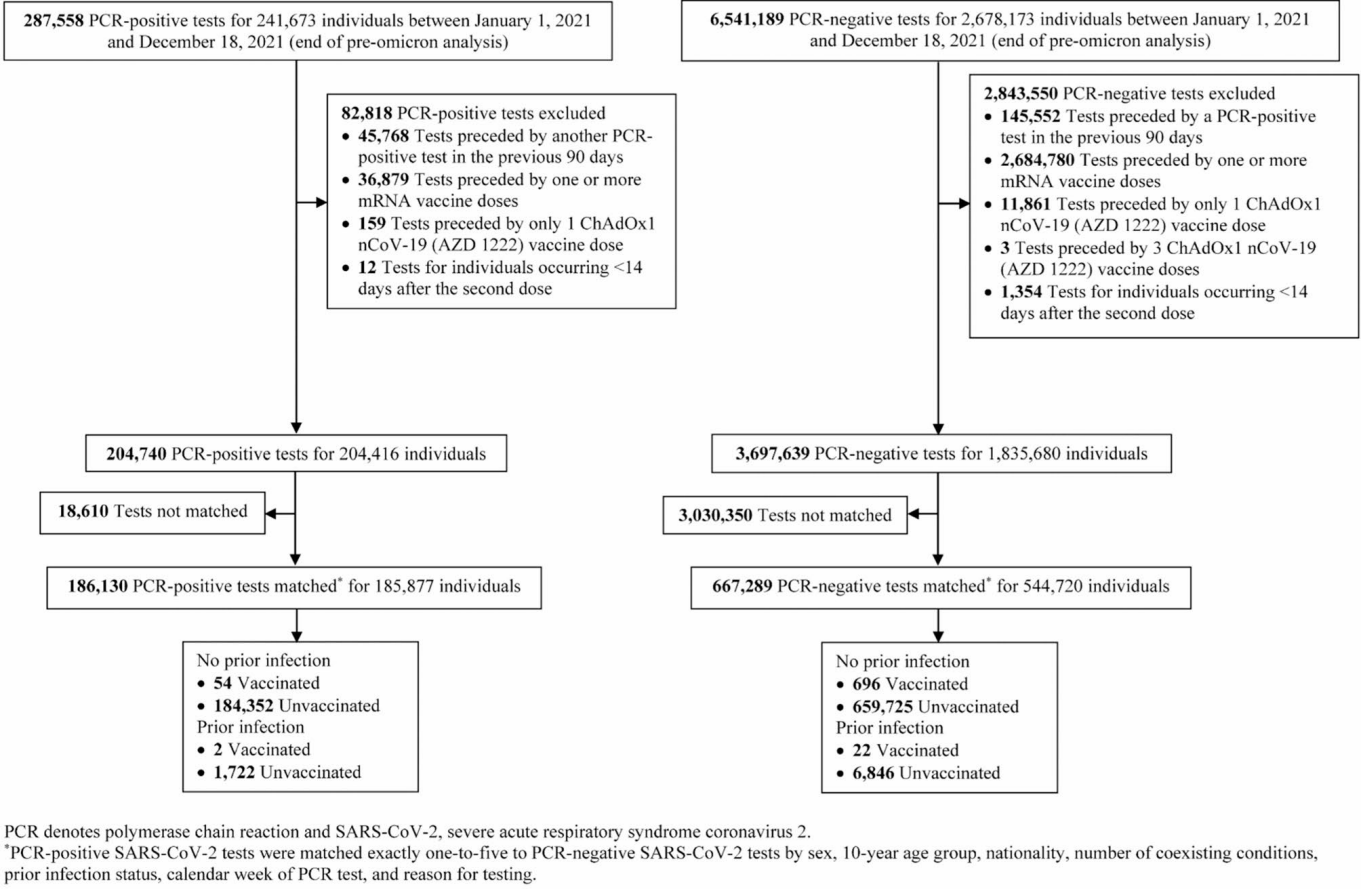
Flowchart describing the study population selection process for investigating ChAdOx1 nCoV-19 (Vaxzevria) vaccine effectiveness

**Fig. 3:**
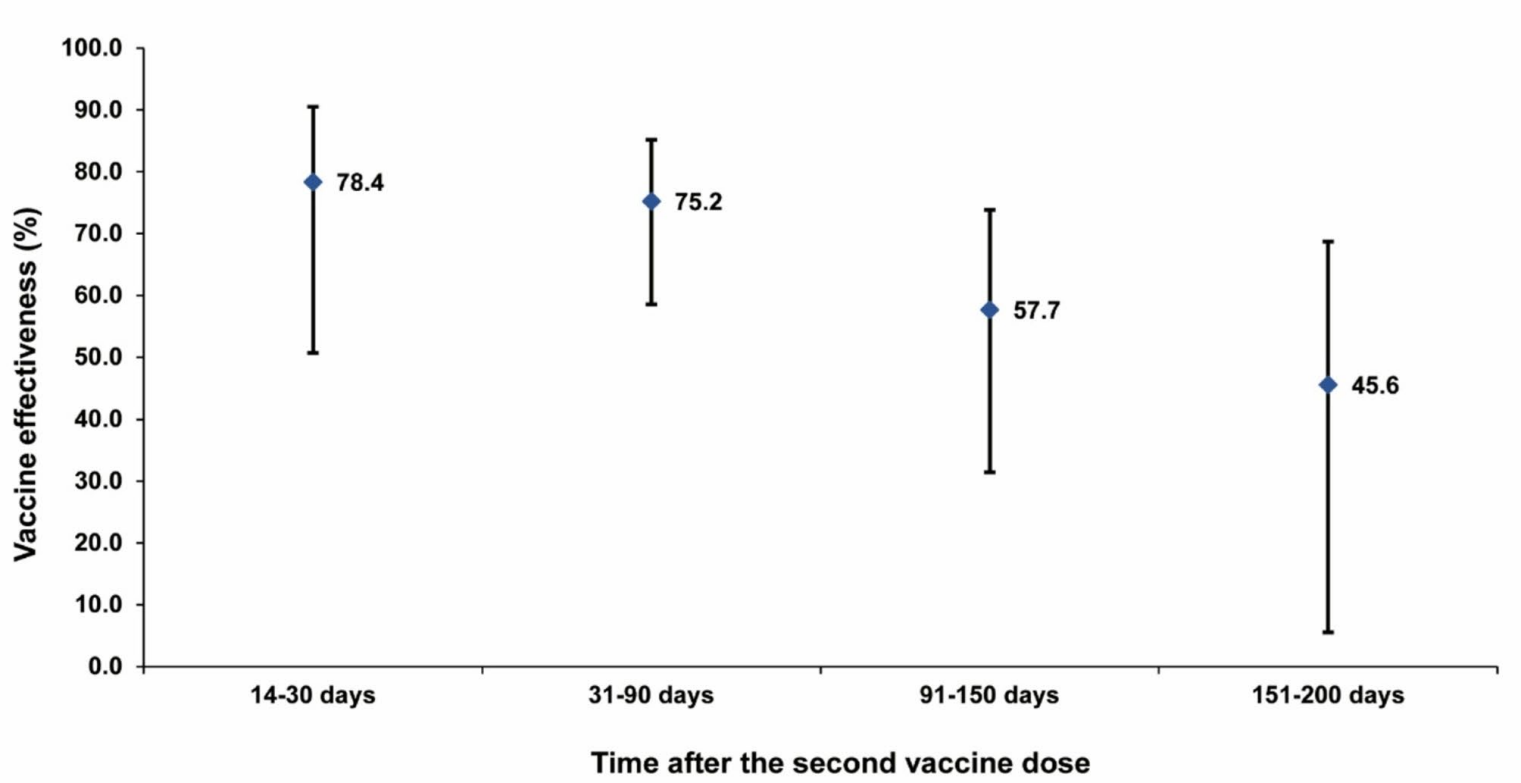
Effectiveness of the ChAdOx1 nCoV-19 (Vaxzevria) vaccine against infection by time since the second vaccine dose. Error bars indicate the 95% confidence intervals for the estimated vaccine effectiveness (blue markers)

Figure 2 outlines the selection process for the study population. Of 287,558 PCR-positive tests from 241,673 individuals, 82,818 were excluded due to recent prior infection, receipt of mRNA vaccines, having only one ChAdOx1 nCoV-19 dose, or insufficient time since the second dose, leaving 204,740 eligible tests. Of these, 186,130 were successfully matched to controls. Among 6,541,189 PCR-negative tests from 2,678,173 individuals, 2,843,550 were excluded for similar reasons, resulting in 3,697,639 eligible tests, of which 667,289 were matched to cases. The final matched dataset included 186,130 PCR-positive tests from 185,877 individuals and 667,289 PCR-negative tests from 544,720 individuals.

Table 1 presents the characteristics of cases and controls in both unmatched and matched samples. As the study was conducted on Qatar’s population, the study population reflects the country’s internationally diverse, yet predominantly young and male demographic profile.

**Table 1:** Characteristics of cases and controls in samples used to estimate the effectiveness of the ChAdOx1 nCoV-19 (Vaxzevria) vaccine against SARS-CoV-2 infection and against severe COVID-19

Characteristics	Unmatched analysis			Matched analysis		
	Cases*	Controls*	SMD^†^	Cases*	Controls*	SMD^†^
	*N* = 204,740	*N* = 3,697,639		*N* = 186,130	*N* = 667,289	
Median age (IQR)—years	32 (24–39)	30 (21–38)	0.10^‡^	31 (23–38)	31 (23–37)	0.06^‡^
Age group—no. (%)						
< 10 years	21,312 (10.4)	526,774 (14.2)	0.15	19,566 (10.5)	73,488 (11.0)	0.08
10–19 years	18,685 (9.1)	324,387 (8.8)		16,826 (9.0)	59,861 (9.0)	
20–29 years	45,903 (22.4)	900,034 (24.3)		44,107 (23.7)	171,397 (25.7)	
30–39 years	69,549 (34.0)	1,133,095 (30.6)		65,715 (35.3)	236,580 (35.5)	
40–49 years	34,884 (17.0)	537,270 (14.5)		30,208 (16.2)	98,418 (14.7)	
50–59 years	11,129 (5.4)	202,720 (5.5)		8,175 (4.4)	23,849 (3.6)	
60–69 years	2,499 (1.2)	57,710 (1.6)		1,273 (0.7)	3,153 (0.5)	
70 + years	779 (0.4)	15,649 (0.4)		260 (0.1)	543 (0.1)	
Sex						
Male	140,390 (68.6)	2,655,156 (71.8)	0.07	129,004 (69.3)	468,425 (70.2)	0.02
Female	64,350 (31.4)	1,042,483 (28.2)		57,126 (30.7)	198,864 (29.8)	
Nationality^§^						
Bangladeshi	14,967 (7.3)	188,752 (5.1)	0.27	13,820 (7.4)	45,961 (6.9)	0.09
Egyptian	11,084 (5.4)	182,410 (4.9)		10,019 (5.4)	32,516 (4.9)	
Filipino	22,658 (11.1)	215,701 (5.8)		21,234 (11.4)	73,160 (11.0)	
Indian	57,089 (27.9)	1,192,425 (32.2)		54,807 (29.4)	208,913 (31.3)	
Nepalese	18,169 (8.9)	296,022 (8.0)		16,746 (9.0)	60,174 (9.0)	
Pakistani	10,242 (5.0)	208,323 (5.6)		9,269 (5.0)	34,947 (5.2)	
Qatari	21,653 (10.6)	401,165 (10.8)		21,035 (11.3)	86,787 (13.0)	
Sri Lankan	6,726 (3.3)	75,055 (2.0)		5,922 (3.2)	18,452 (2.8)	
Sudanese	5,235 (2.6)	73,557 (2.0)		4,534 (2.4)	14,924 (2.2)	
Other nationalities^¶^	36,917 (18.0)	864,229 (23.4)		28,744 (15.4)	91,455 (13.7)	
Coexisting conditions**						
0	168,285 (82.2)	3,239,872 (87.6)	0.16	159,146 (85.5)	587,477 (88.0)	0.08
1	22,250 (10.9)	298,426 (8.1)		18,250 (9.8)	57,392 (8.6)	
2	8,465 (4.1)	94,203 (2.5)		5,854 (3.1)	15,987 (2.4)	
3	2,929 (1.4)	32,160 (0.9)		1,591 (0.9)	3,814 (0.6)	
4	1,394 (0.7)	15,626 (0.4)		651 (0.3)	1,322 (0.2)	
5	769 (0.4)	8,458 (0.2)		307 (0.2)	591 (0.1)	
6+	648 (0.3)	8,894 (0.2)		331 (0.2)	706 (0.1)	
Prior infection status						
No prior infection	202,636 (99.0)	3,512,961 (95.0)	0.23	184,406 (99.1)	660,421 (99.0)	0.01
Prior infection	2,104 (1.0)	184,678 (5.0)		1,724 (0.9)	6,868 (1.0)	
Reason for testing						
Clinical suspicion	60,732 (29.7)	209,840 (5.7)	1.06	50,286 (27.0)	107,634 (16.1)	0.32
Contact tracing	25,067 (12.2)	147,249 (4.0)		22,251 (12.0)	74,766 (11.2)	
Survey	34,071 (16.6)	555,883 (15.0)		32,131 (17.3)	134,905 (20.2)	
Port of entry	44,159 (21.6)	1,833,716 (49.6)		43,596 (23.4)	213,969 (32.1)	
Individual request	14,875 (7.3)	182,759 (4.9)		13,989 (7.5)	59,733 (9.0)	
Healthcare routine testing	18,627 (9.1)	129,120 (3.5)		17,493 (9.4)	47,823 (7.2)	
Pre-travel	6,552 (3.2)	629,773 (17.0)		6,150 (3.3)	28,005 (4.2)	
Other	657 (0.3)	9,299 (0.3)		234 (0.1)	454 (0.1)	

*IQR* denotes interquartile range, *COVID-19* coronavirus disease 2019, *PCR* polymerase chain reaction, *SARS-CoV-2* severe acute respiratory syndrome coronavirus 2, and *SMD* standardized mean difference

*Cases (PCR-positive tests) and controls (PCR-negative tests) were matched exactly one-to-five by sex, 10-year age group, nationality, number of coexisting conditions, prior infection status, calendar week of PCR test, and reason for testing

^†^SMD quantifies covariate balance between groups and is calculated as the difference in the covariate means divided by the pooled standard deviation. An SMD ≤ 0.1 is typically interpreted as indicating adequate balance. However, in matched samples with variable matching ratios (e.g., one to five controls per case), SMD values may exceed 0.1 even when exact matching on covariate values is applied and cases and controls are fully balanced by design. This apparent imbalance reflects the sensitivity of SMD to differences in within-group variance introduced by variable matching ratios, rather than true imbalance in covariate distributions

^‡^SMD is for the mean difference between groups divided by the pooled standard deviation

^§^Nationalities were chosen to represent the most populous groups in Qatar

^¶^These comprise up to 183 other nationalities in Qatar in the unmatched and 123 nationalities in the matched analysis

**Table 2:** Effectiveness of the ChAdOx1 nCoV-19 (Vaxzevria) vaccine against (A) any infection, (B) symptomatic infection, and (C) severe, critical or fatal COVID-19

Effectiveness	Cases*		Controls*		Effectiveness^†^ in % (95% CI)^‡^
	Vaccinated (*n*)	Unvaccinated (*n*)	Vaccinated (*n*)	Unvaccinated (*n*)	
(A) Any infection	56	186,074	718	666,571	66.0 (55.1 to 74.3)
Variant					
Beta	0	105,941	12	321,306	100 (64.0 to 100)^¶^
Delta	56	35,497	704	161,675	65.3 (54.2 to 73.8)
Time after second dose					
14–30 days	6	186,063	125	666,349	78.4 (50.7 to 90.5)
31–90 days	16	186,066	275	666,387	75.2 (58.6 to 85.2)
91–150 days	19	186,068	192	666,412	57.7 (31.4 to 73.9)
151–200 days	15	186,074	122	666,571	45.6 (5.5 to 68.7)
Prior infection status					
No prior infection	54	184,352	696	659,725	66.0 (54.8 to 74.4)
Prior infection	2	1,722	22	6,846	66.5 (-33.7 to 92.6)
Age					
< 50 years	54	176,368	691	639,053	65.5 (54.2 to 74.0)
≥ 50 years	2	9,706	27	27,518	75.6 (-5.0 to 94.3)
Clinical vulnerability to severe COVID-19					
Less clinically vulnerable	54	170,043	683	621,994	64.6 (53.0 to 73.4)
More clinically vulnerable	2	16,031	35	44,577	84.0 (32.0 to 96.2)
(B) Symptomatic infection^II^	8	50,278	121	107,513	73.0 (44.1 to 87.0)
(C) Severe, critical, or fatal COVID-19^§^	0	4,785	9	18,305	100 (49.3 to 100)^¶^

*CI* denotes confidence interval, *COVID-19* coronavirus disease 2019, and *SARS-CoV-2* severe acute respiratory syndrome coronavirus 2

*Cases (PCR-positive tests) and controls (PCR-negative tests) were matched exactly one-to-five by sex, 10-year age group, nationality, number of coexisting conditions, prior infection status, calendar week of PCR test, and reason for testing

^†^Vaccine effectiveness was estimated using the test-negative, case–control study design

^‡^CIs were not adjusted for multiplicity and thus should not be used to infer definitive differences between different groups

^§^Severity, criticality, and fatality were defined according to the World Health Organization guidelines

^II^A symptomatic infection was defined as a SARS-CoV-2 PCR-positive test conducted because of clinical suspicion due to presence of symptoms compatible with a respiratory tract infection

^¶^The 95% CI was estimated using McNemar’s test because of zero vaccinations among cases

### Two-dose primary series vaccine effectiveness against infection and severe COVID-19

The overall effectiveness of the ChAdOx1 nCoV-19 primary series in preventing SARS-CoV-2 infection, irrespective of symptoms, was estimated at 66.0% (95% CI, 55.1–74.3%) (Table 2). Effectiveness against symptomatic infection was higher, estimated at 73.0% (95% CI, 44.1–87.0%). Effectiveness against severe, critical, or fatal COVID-19 was estimated at 100% (95% CI, 49.3–100%), with no vaccinated individuals progressing to severe, critical, or fatal disease following infection.

Effectiveness against Beta variant infection, irrespective of symptoms, was estimated at 100% (95% CI, 64.0–100%), while effectiveness against Delta variant infection was 65.3% (95% CI, 54.2–73.8%). In the Beta variant analysis, the median date of the second dose was March 13, 2021 (IQR, February 24–April 9, 2021), whereas in the Delta variant analysis, it was June 28, 2021 (IQR, June 17–June 29, 2021). The median interval between the second dose and the PCR test was 53 days (IQR, 21–62.5 days) in the Beta variant analysis and 81 days (IQR, 39–130.5 days) in the Delta variant analysis.

Effectiveness against any infection, regardless of variant, peaked at 78.4% (95% CI, 50.7–90.5%) during the 14–30 days following the second dose (Fig. 3). It remained relatively high at 75.2% (95% CI, 58.6–85.2%) between 31 and 90 days post-vaccination but declined to 57.7% (95% CI, 31.4–73.9%) during days 91–150. Thereafter, effectiveness further waned to 45.6% (95% CI, 5.5–68.7%) between days 151 and 200 following the second dose.

There was no evidence that vaccine effectiveness differed by prior infection status, age group, or clinical vulnerability status (Table 2). However, the estimated effectiveness in several of these subgroups was associated with wide 95% CIs, limiting the ability to make statistically precise comparisons.

### Additional analysis: one-dose vaccine effectiveness against infection and severe COVID-19

The overall effectiveness of a single dose of the ChAdOx1 nCoV-19 vaccine was estimated at 59.9% (95% CI, 51.0–67.3%) against any SARS-CoV-2 infection, 78.4% (95% CI, 60.9–88.0%) against symptomatic infection, and 100% (95% CI, 88.9–100%) against severe, critical, or fatal COVID-19, with no severe cases reported among vaccinated individuals. Variant-specific effectiveness was estimated at 65.0% (95% CI, 49.7–75.6%) against Beta variant infection and 55.9% (95% CI, 43.8–65.5%) against Delta variant infection, irrespective of symptoms.

## Discussion

The findings demonstrate substantial protection conferred by the ChAdOx1 nCoV-19 primary series, with overall effectiveness of 66.0% against infection during periods dominated by the Beta and Delta variants. Notably, a single dose also provided considerable protection, with effectiveness estimated at 59.9%. Protection peaked within the first three months following the second dose but declined to below 50% after 150 days.

This waning of effectiveness aligns with evidence of declining protection over time for both ChAdOx1 nCoV-19 [58–61] and mRNA vaccines [17, 48], underscoring the need for booster doses to sustain protection. The vaccine also provided strong protection against severe, critical, or fatal COVID-19, with no severe cases reported among vaccinated individuals during the study period. This reinforces the vaccine’s role in reducing hospitalizations and deaths, consistent with findings from other studies [59, 62–67], and supports its broad protective benefits across population groups and clinical presentations.

Vaccine effectiveness against the Delta variant was estimated at 65%, consistent with vaccine efficacy reported against this variant in an RCT [10] and with findings from real-world effectiveness studies conducted in Canada [63], the Czech Republic [59], Finland [58], India [68], the Netherlands [69, 70], Scotland [64], Spain [71], Thailand [60], and the UK [72, 73].

Importantly, this study, to our knowledge, provided the first statistically meaningful estimate of the effectiveness of the ChAdOx1 nCoV-19 vaccine against the Beta variant [1], demonstrating strong protection. No infection cases were identified among vaccinated individuals, and the estimated minimum effectiveness was 64%. Another estimate from Canada also indicated strong protection against Beta, although the 95% CI was very wide [62].

This finding contrasts with results from the RCT conducted in South Africa, which reported negligible efficacy against the Beta variant, accompanied by a fairly wide 95% CI [9, 10]. In response to that trial, several countries where Beta was circulating discontinued use of this vaccine, despite global vaccine shortages at the time [74]. Reports of rare blood clotting events associated with thromboembolic and thrombocytopenic complications also contributed to the decision [13, 14]. The present findings suggest that this decision may have been suboptimal and potentially premature, limiting access to an affordable vaccine that could have provided protection against infection, reduced severe disease outcomes, and averted deaths during a period of high transmission and unmet vaccination need [14].

Notably, a similar discrepancy was observed for mRNA vaccines and natural infection in relation to protection against infection with the Beta variant. Molecular-level evidence initially suggested that Beta might evade both vaccine-induced and naturally acquired immunity [75, 76], but population-level data later demonstrated that the impact of Beta mutations on protection conferred by mRNA vaccination and prior infection was modest [12, 15, 16].

This study has limitations. Although it included a large number of PCR tests, the number of individuals vaccinated with ChAdOx1 nCoV-19 in Qatar was relatively small due to the vaccine’s early discontinuation. This limited the feasibility and statistical power for some subgroup analyses, particularly for assessing effectiveness over time and against symptomatic infection and severe COVID-19 by variant type. It also constrained the feasibility of relevant analyses, such as evaluating the impact of dosing intervals on vaccine effectiveness. Furthermore, the findings may have reduced generalizability to settings where this vaccine was more widely used.

Variant classification was based on periods of variant dominance rather than genomic sequencing or genotyping of all infections. There also was a difference of nearly one month in the median interval between the second dose and the PCR test in the Beta versus Delta analyses, which may have contributed—albeit modestly—to the observed differences in effectiveness between the two variants. A meaningful analysis of post-vaccination clotting events was not feasible due to the limited number of cases. Only sixteen stroke-related events of any type were reported among individuals who had ever received a dose of ChAdOx1 nCoV-19 [77], a number far too small to allow for a reliable assessment of any potential causal relationship.

COVID-19 vaccination in Qatar was offered free of charge to all citizens and residents through the public healthcare system [11], with prioritization based on factors such as age, chronic conditions, and occupation [17]. The ChAdOx1 nCoV-19 vaccine was available for only a short, specific period before being discontinued and was primarily administered through field-based mass campaigns. As a result, recipients of the ChAdOx1 nCoV-19 vaccine and those who received mRNA vaccines may differ. However, rigorous matching was applied in this study to adjust for potential confounding factors. Moreover, since the analysis estimates the biological effect of vaccination, which is not expected to vary by socioeconomic factors, differences between recipients of different vaccines should not meaningfully impact the estimated outcomes.

This study was conducted in a unique national population that differs substantially from those of most other countries. Qatar has a distinctly young and diverse demographic profile, with only 9% of residents aged ≥ 50 years and 89% comprising expatriates from over 150 countries [29, 78]. Additionally, more than two-thirds of the population are male, primarily migrant workers typically employed in large infrastructure projects [29, 31, 44–47]. As such, the extent to which the study findings can be generalized to other populations remains uncertain.

The observational test-negative design can be subject to potential bias, including from unmeasured differences in test-seeking behavior or changes in testing patterns. Most healthcare data were captured electronically, but misclassification bias remains a concern in real-world data. Such bias may arise from inaccuracies in exposure (e.g., missing vaccination records), covariates (e.g., comorbidities), or outcomes (e.g., hospitalizations outside Qatar), and may be non-differential across study groups.

Matching in case-control studies helps reduce confounding but may not eliminate bias and may introduce selection bias [79]. Although matching included key variables such as age, sex, and nationality, other factors like geographic location or occupation could not be accounted for due to data limitations. However, Qatar’s city-state structure supports relatively uniform infection risk across neighborhoods, and age, sex, and nationality serve as reliable proxies for socio-economic status in this population [29, 44–47]. Matching on these variables may have partially controlled for unmeasured differences in exposure risk, including occupation. This approach has been validated in prior studies using various designs and control groups to test for null effects, with findings supporting its robustness [11, 16, 17, 48, 80].

This study has strengths. It is the first to provide an estimate of the real-world effectiveness of the ChAdOx1 nCoV-19 vaccine against the Beta variant. The analysis was conducted at the national level, encompassing a diverse population with a wide range of national backgrounds. Findings were based on comprehensive, validated national databases established through prior vaccine effectiveness studies, with infections confirmed by PCR testing. The use of an integrated digital health information platform enabled robust data capture on potential confounders and facilitated rigorous matching based on socio-demographic characteristics and a set of health indicators. Controls were drawn from the national population using exact matching, ensuring precise pairing with cases.

In conclusion, this study provides real-world evidence of the effectiveness of the ChAdOx1 nCoV-19 vaccine, including against the Beta variant, for which prior data were limited and inconclusive. The findings affirm the vaccine’s substantial protection against SARS-CoV-2 infection and its strong protection against severe COVID-19 outcomes, including during periods dominated by the Beta and Delta variants. These results contribute to a more complete understanding of the vaccine’s performance across diverse settings and variants, challenge early assumptions, and underscore the value of leveraging national data systems to inform policy decisions.

## Supplementary Information

Below is the link to the electronic supplementary material.

Supplementary Material 1

## Data Availability

The dataset of this study is the property of the Qatar Ministry of Public Health and was provided to the researchers through a restricted-access agreement that prohibits sharing the dataset with third parties or making it publicly available. Access to the data is restricted to preserve the confidentiality of patient information and was granted to researchers for research purposes only. Access can be obtained through a direct application for data access to Her Excellency the Minister of Public Health (https://www.moph.gov.qa/english/OurServices/eservices/Pages/Governmental-HealthCommunication-Center.aspx). Data were available to authors through.csv files where information has been downloaded from the CERNER database system (no links/accession codes were available to authors). All proposed research must obtain the necessary ethical approvals. Commercial use of the data is strictly prohibited. Requests for access are assessed by the Ministry of Public Health in Qatar, and approval is granted at its discretion. In compliance with data privacy laws and the data-sharing agreement with the Ministry of Public Health in Qatar, no datasets, whether raw or de-identified, can be publicly released by the researchers. However, aggregate data that do not compromise individual privacy are included within the manuscript and supplementary materials. This ensures transparency of the research findings and supports the reproducibility of results while maintaining compliance with legal requirements.
