# Supplementary material for: Effectiveness of ChAdOx1 nCoV-19 (Vaxzevria) primary series vaccine against SARS-CoV-2 beta and delta variants: a nationwide study
Source: BMC Infect Dis. 2025 Aug 17;25:1028. doi: 10.1186/s12879-025-11410-7 (PMC12358068; doi:10.1186/s12879-025-11410-7)
Supplement: Supplementary file 1 — Supplementary Material 1 [file 12879_2025_11410_MOESM1_ESM.docx]

**Supplementary Appendix**

**Table of Contents**

[**Supplementary Fig. 1. Daily count of newly diagnosed SARS-CoV-2 infections up to the end of the study, between February 28, 2020 and December 18, 2021.** 2](#_Toc203561978)

[**Supplementary Section 1. Study population and data sources** 3](#_Toc203561979)

[**Supplementary Section 2. Laboratory methods and variant ascertainment** 6](#_Toc203561980)

[**Real-time reverse-transcription polymerase chain reaction testing** 6](#_Toc203561981)

[**Classification of infections by variant type** 6](#_Toc203561982)

[**Supplementary Section 3. Classification of coexisting conditions** 7](#_Toc203561983)

[**Supplementary Section 4. COVID-19 severity, criticality, and fatality classification** 9](#_Toc203561984)

[**Severe COVID-19** 9](#_Toc203561985)

[**Critical COVID-19** 9](#_Toc203561986)

[**Fatal COVID-19** 10](#_Toc203561987)

[**Supplementary Table 1.** **Strengthening the Reporting of Observational Studies in Epidemiology (STROBE) checklist for case-control studies.** 11](#_Toc203561988)

[**References** 13](#_Toc203561989)

#

# **Supplementary Fig. 1.** **Daily count of newly diagnosed SARS-CoV-2 infections up to the end of the study, between February 28, 2020 and December 18, 2021.**


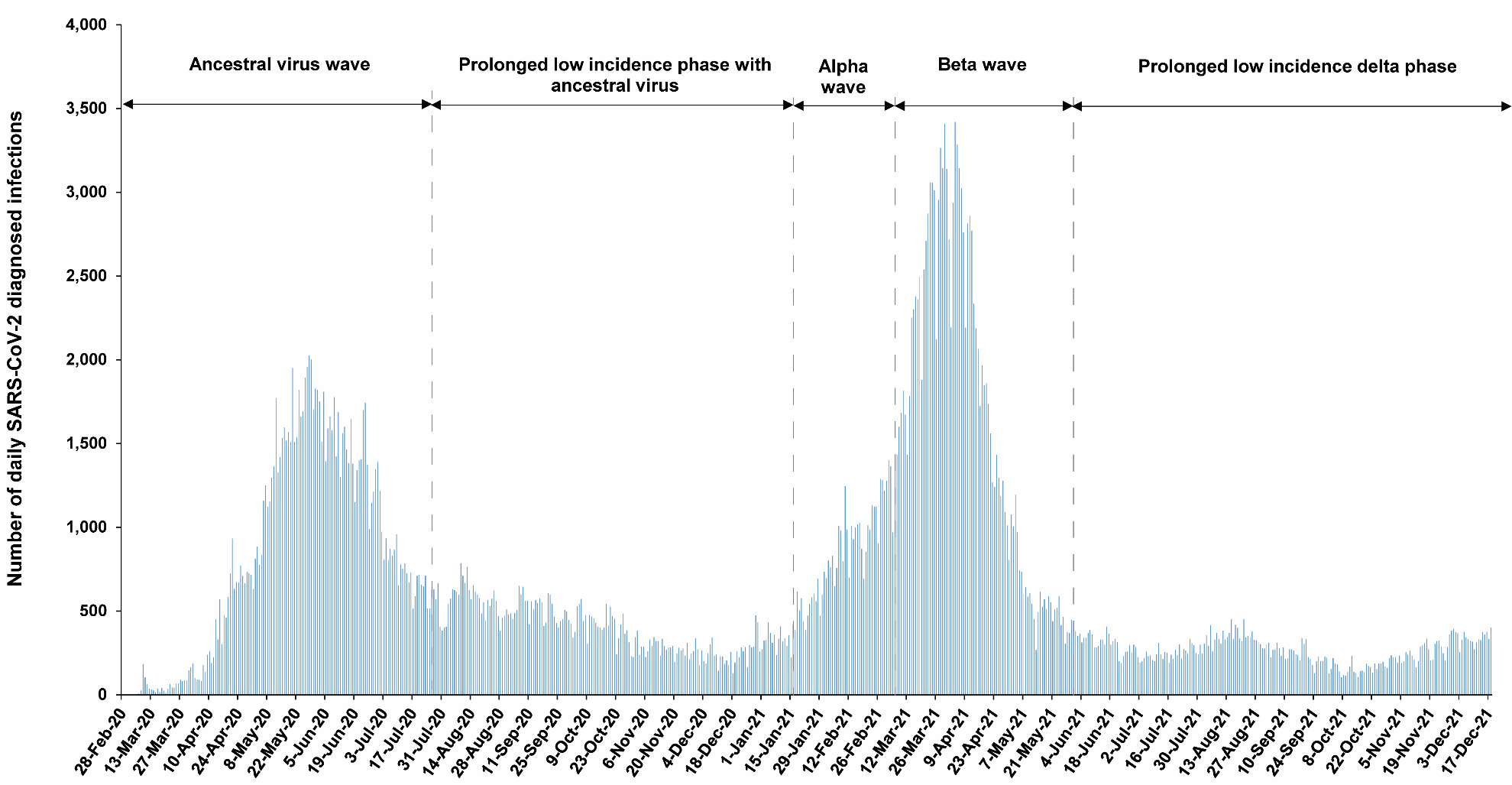


#

# **Supplementary Section 1. Study population and data sources**

Qatar’s national and universal public healthcare system uses the Cerner Millenium electronic medical record (EMR) system to track all the public healthcare encounters of each individual in the country, including all citizens and residents registered in the national and universal public healthcare system. Registration in the public healthcare system is mandatory for citizens and residents.

The databases analyzed in this study are data-extract downloads from the national EMR database that have been implemented on a regular weekly schedule since the onset of pandemic by the Business Intelligence Unit at Hamad Medical Corporation (HMC). HMC is the national public healthcare provider in Qatar. At every download, all severe acute respiratory syndrome coronavirus 2 (SARS-CoV-2) tests, coronavirus disease 2019 (COVID-19) vaccinations, hospitalizations related to COVID-19, and all death records regardless of cause are provided to the authors through .csv files. These databases have been analyzed throughout the pandemic not only for study-related purposes, but also to provide policymakers with summary data and analytics to inform the national response.

Every health encounter in the national EMR is linked to an individual through the HMC Number, which serves as a unique identifier that links all records for this individual at the national level. Databases were merged and analyzed using the HMC Number to link all records pertaining to testing, vaccinations, hospitalizations, and deaths. All deaths in Qatar are recorded by the public healthcare system. COVID-19-related healthcare was provided exclusively in the public healthcare system. COVID-19 vaccination was also provided only through the public healthcare system. These health records were tracked throughout the COVID-19 pandemic using the national EMR system. This system has been implemented in 2013, before the onset of the pandemic. This pre-established system ensured that we had access to comprehensive health records related to this study for both citizens and residents throughout the entire pandemic, allowing us to follow each person over time.

Demographic details for every HMC Number (individual) such as sex, age, and nationality are collected upon issuing of the universal health card, based on the Qatar Identity Card, which is a mandatory requirement by the Ministry of Interior to every citizen and resident in the country. Data extraction from the Qatar Identity Card to the digital health platform is performed electronically through scanning techniques.

SARS-CoV-2 testing in any facility in Qatar is tracked nationally in one database, the national testing database. This database covers all testing throughout the country, whether in public or private facilities. Every polymerase chain reaction (PCR) test conducted in Qatar, regardless of location or setting, is classified on the basis of symptoms and the reason for testing, such as the presence of clinical symptoms, contact tracing, participation in surveys or random testing campaigns, individual requests for testing, routine healthcare testing, pre-travel requirements, at the point of entry into the country, or any other relevant reasons for testing.

Before November 1, 2022, SARS-CoV-2 testing in Qatar was performed extensively with about 5% of the population tested every week [1]. Based on the distribution of the reason for testing up to November 1, 2022, most of the tests in Qatar were conducted for routine reasons, such as travel-related purposes, and about 75% of infections were diagnosed not because of presence of symptoms [1, 2]. This study factored all SARS-CoV-2-related testing included in the national testing database over the duration of the study.

Qatar launched its COVID-19 vaccination program in December 2020, employing mRNA vaccines and prioritizing individuals based on coexisting conditions and age criteria [2, 3]. COVID-19 vaccination was provided free of charge, regardless of citizenship or residency status, and was nationally tracked [2, 3].

Qatar has unusually young, diverse demographics, in that only 9% of its residents are ≥50 years of age, and 89% are expatriates from over 150 countries [2, 4]. Further descriptions of the study population and these national databases were reported previously [1, 2, 5-9].

# **Supplementary Section 2. Laboratory methods and variant ascertainment**

## **Real-time reverse-transcription polymerase chain reaction testing**

Nasopharyngeal and/or oropharyngeal swabs were collected for PCR testing and placed in Universal Transport Medium (UTM). Aliquots of UTM were: 1) extracted on KingFisher Flex (Thermo Fisher Scientific, USA), MGISP-960 (MGI, China), or ExiPrep 96 Lite (Bioneer, South Korea) followed by testing with real-time reverse-transcription PCR (RT-qPCR) using TaqPath COVID-19 Combo Kits (Thermo Fisher Scientific, USA) on an ABI 7500 FAST (Thermo Fisher Scientific, USA); 2) tested directly on the Cepheid GeneXpert system using the Xpert Xpress SARS-CoV-2 (Cepheid, USA); or 3) loaded directly into a Roche cobas 6800 system and assayed with the cobas SARS-CoV-2 Test (Roche, Switzerland). The first assay targets the viral S, N, and ORF1ab gene regions. The second targets the viral N and E-gene regions, and the third targets the ORF1ab and E-gene regions.

All PCR testing was conducted at the HMC Central Laboratory or Sidra Medicine Laboratory, following standardized protocols.

## **Classification of infections by variant type**

Surveillance for SARS-CoV-2 variants in Qatar is based on viral genome sequencing and multiplex RT-qPCR variant screening [10] of weekly collected random positive clinical samples [11-16], complemented by deep sequencing of wastewater samples [13, 17, 18]. Further details on the viral genome sequencing and multiplex RT-qPCR variant screening throughout the SARS-CoV-2 waves in Qatar can be found in previous publications [1, 11-16, 19-26].

# **Supplementary Section 3. Classification of coexisting conditions**

The classification of patients and their coexisting conditions was carried out by an independent medical team appointed by the Ministry of Public Health. This process was part of routine needs assessment exercises aimed at planning healthcare use, utilization, and resource allocation.

As Qatar’s medical coding systems evolved over time, incorporating different versions of ICD codes as well as SNOMED codes, the medical team developed mappings to harmonize these codes and ensure consistent categorization of coexisting conditions. The study investigators did not have direct access to the detailed procedures, documentation, or specific codes and mappings used by this independent team. Instead, the finalized database of patients and their coexisting conditions was provided to the study investigators exclusively for research purposes.

Coexisting conditions were ascertained and classified based on the mapped codes for the conditions, as recorded in the electronic health record encounters of each individual in the national EMR database that includes all citizens and residents registered in the national and universal public healthcare system. The public healthcare system provides healthcare to the entire resident population of Qatar free of charge or at heavily subsidized costs, including prescription drugs. With the mass expansion of this sector in recent years, facilities have been built to cater to specific needs of subpopulations. For example, tens of facilities have been built, including clinics and hospitals, in localities with high density of craft and manual workers [27].

All encounters for each individual were analyzed to determine the coexisting-condition classification for that individual. The national EMR database includes encounters starting from 2013, when this system was launched in Qatar. Any individual who had at least one encounter with a specific coexisting-condition diagnosis since 2013 was classified as having that coexisting condition. Individuals who do not have records of coexisting-condition encounters in the public healthcare system were classified as having no coexisting conditions.

The classification of coexisting conditions spanned the following conditions: 1) Behchet’s disease, 2) cancer, 3) cardiovascular diseases, 4) infectious and parasitic diseases, 5) Crohn’s disease, 6) chronic kidney disease (CKD), 7) chronic liver disease (CLD), 8) chronic lung disease, 9) congenital malformations, deformations and chromosomal abnormalities, 10) diseases of the blood and blood-forming organs and certain disorders involving the immune mechanism, 11) diseases of the ear and mastoid process, 12) deep vein thrombosis (DVT), 13) dermatitis, 14) diabetes mellitus, 15) diseases of the circulatory system, 16) diseases of the digestive system, 17) diseases of the eye and adnex, 18) diseases of the genitourinary system, 19) diseases of the musculoskeletal system and connective tissue, 20) diseases of the nervous system, 21) diseases of the respiratory system, 22) diseases of the skin and subcutaneous tissue, 23) endocrine, nutritional and metabolic diseases, 24) gingivitis, 25) hypertension, 26) injury, poisoning and certain other consequences of external causes, 27) mental and behavioral disorders, 28) neoplasms, 29 periodontitis, 30) pregnancy, childbirth and the puerperium, 31) pulmonary tuberculosis, 32) rheumatoid arthritis, 33) Sjogren’s syndrome, 34) stroke or neural conditions, 35) symptoms, signs and abnormal clinical and laboratory findings, not elsewhere classified, 36) systemic lupus erythematosus, 37) systemic sclerosis, 38) organ transplant, and 39) other unspecified factors influencing health status and contact with health services.

# **Supplementary Section 4. COVID-19 severity, criticality, and fatality classification**

Classification of COVID-19 case severity (acute-care hospitalizations) [28], criticality (intensive-care-unit hospitalizations) [28], and fatality [29] followed World Health Organization (WHO) guidelines. Assessments were made by trained medical personnel independent of study investigators and using individual chart reviews, as part of a national protocol applied to every hospitalized COVID-19 patient. Each hospitalized COVID-19 patient underwent an infection severity assessment every three days until discharge or death. We classified individuals who progressed to severe, critical, or fatal COVID-19 between the time of the documented infection and the end of the study based on their worst outcome, starting with death [29], followed by critical disease [28], and then severe disease [28].

## **Severe COVID-19**

Severe COVID-19 disease was defined per WHO classification as a SARS-CoV-2 infected person with “oxygen saturation of <90% on room air, and/or respiratory rate of >30 breaths/minute in adults and children >5 years old (or ≥60 breaths/minute in children <2 months old or ≥50 breaths/minute in children 2-11 months old or ≥40 breaths/minute in children 1–5 years old), and/or signs of severe respiratory distress (accessory muscle use and inability to complete full sentences, and, in children, very severe chest wall indrawing, grunting, central cyanosis, or presence of any other general danger signs)” [28]. Detailed WHO criteria for classifying SARS-CoV-2 infection severity can be found in the WHO technical report [28].

## **Critical COVID-19**

Critical COVID-19 disease was defined per WHO classification as a SARS-CoV-2 infected person with “acute respiratory distress syndrome, sepsis, septic shock, or other conditions that would normally require the provision of life sustaining therapies such as mechanical ventilation (invasive or non-invasive) or vasopressor therapy” [28]. Detailed WHO criteria for classifying SARS-CoV-2 infection criticality can be found in the WHO technical report [28].

## **Fatal COVID-19**

COVID-19 death was defined per WHO classification as “a death resulting from a clinically compatible illness, in a probable or confirmed COVID-19 case, unless there is a clear alternative cause of death that cannot be related to COVID-19 disease (e.g. trauma). There should be no period of complete recovery from COVID-19 between illness and death. A death due to COVID-19 may not be attributed to another disease (e.g. cancer) and should be counted independently of preexisting conditions that are suspected of triggering a severe course of COVID-19” [29]. Detailed WHO criteria for classifying COVID-19 death can be found in the WHO technical report [29].

# **Supplementary Table 1.** **Strengthening the Reporting of Observational Studies in Epidemiology (STROBE) checklist for case-control studies.**

|  | Item No | Recommendation | Main text page |
| --- | --- | --- | --- |
| **Title and abstract** | 1 | (*a*) Indicate the study’s design with a commonly used term in the title or the abstract | Abstract |
|  |  | (*b*) Provide in the abstract an informative and balanced summary of what was done and what was found | Abstract |
| Introduction | | |  |
| Background/rationale | 2 | Explain the scientific background and rationale for the investigation being reported | Introduction |
| Objectives | 3 | State specific objectives, including any prespecified hypotheses | Introduction |
| Methods | | |  |
| Study design | 4 | Present key elements of study design | Methods (‘Study design’) |
| Setting | 5 | Describe the setting, locations, and relevant dates, including periods of recruitment, exposure, follow-up, and data collection | Methods (‘Study population and data sources’, ‘Study design’, & ‘One-dose vaccine effectiveness against infection and severe COVID-19’), Fig. 2, & Supplementary Section 1 |
| Participants | 6 | (*a*) Give the eligibility criteria, and the sources and methods of case ascertainment and control selection. Give the rationale for the choice of cases and controls | Methods (‘Study population and data sources’, ‘Study design’, & ‘One-dose vaccine effectiveness against infection and severe COVID-19’), Fig. 2, & Supplementary Section 1 |
|  |  | (*b*) For matched studies, give matching criteria and the number of controls per case |  |
| Variables | 7 | Clearly define all outcomes, exposures, predictors, potential confounders, and effect modifiers. Give diagnostic criteria, if applicable | Methods (‘Study design’, ‘One-dose vaccine effectiveness against infection and severe COVID-19’, & ‘Statistical Analysis’), Table 1, & Supplementary Sections 1-4 in Supplementary Appendix |
| Data sources/ measurement | 8 | For each variable of interest, give sources of data and details of methods of assessment (measurement). Describe comparability of assessment methods if there is more than one group | Methods (‘Study population and data sources’, ‘Study design’, ‘One-dose vaccine effectiveness against infection and severe COVID-19’, & ‘Statistical analysis’) & Supplementary Sections 1-4 in Supplementary Appendix |
| Bias | 9 | Describe any efforts to address potential sources of bias | Methods (‘Study design’ & ‘Statistical analysis’) |
| Study size | 10 | Explain how the study size was arrived at | Fig. 2 |
| Quantitative variables | 11 | Explain how quantitative variables were handled in the analyses. If applicable, describe which groupings were chosen and why | Methods (‘Study design’ & ‘Statistical analysis’), Table 1, & Supplementary Sections 2-4 in Supplementary Appendix |
| Statistical methods | 12 | (*a*) Describe all statistical methods, including those used to control for confounding | Methods (‘Statistical analysis’) |
|  |  | (*b*) Describe any methods used to examine subgroups and interactions | Methods (‘Study design’, ‘One-dose vaccine effectiveness against infection and severe COVID-19’, & ‘Statistical analysis’) |
|  |  | (*c*) Explain how missing data were addressed | Not applicable, see Methods (‘Study population and data sources’) & Supplementary Section 1 in Supplementary Appendix |
|  |  | (*d*) If applicable, explain how matching of cases and controls was addressed | Methods (‘Study design’, ‘One-dose vaccine effectiveness against infection and severe COVID-19’, & ‘Statistical analysis’) |
|  |  | (*e*) Describe any sensitivity analyses | Not applicable |
| Results | | |  |
| Participants | 13 | (a) Report numbers of individuals at each stage of study—eg numbers potentially eligible, examined for eligibility, confirmed eligible, included in the study, completing follow-up, and analysed | Fig. 2 |
|  |  | (b) Give reasons for non-participation at each stage |  |
|  |  | (c) Consider use of a flow diagram |  |
| Descriptive data | 14 | (a) Give characteristics of study participants (eg demographic, clinical, social) and information on exposures and potential confounders | Results (‘Study population’) & Table 1 |
|  |  | (b) Indicate number of participants with missing data for each variable of interest | Not applicable, see Methods (‘Study population and data sources’) & Supplementary Section 1 in Supplementary Appendix |
| Outcome data | 15 | Report numbers in each exposure category, or summary measures of exposure | Table 2 |
| Main results | 16 | (*a*) Give unadjusted estimates and, if applicable, confounder-adjusted estimates and their precision (eg, 95% confidence interval). Make clear which confounders were adjusted for and why they were included | Results (‘Two-dose primary series vaccine effectiveness against infection and severe COVID-19’), Table 2, & Fig. 3 |
|  |  | (*b*) Report category boundaries when continuous variables were categorized | Table 1 |
|  |  | (*c*) If relevant, consider translating estimates of relative risk into absolute risk for a meaningful time period | Not applicable |
| Other analyses | 17 | Report other analyses done—eg analyses of subgroups and interactions, and sensitivity analyses | Results (‘Additional analysis: One-dose vaccine effectiveness against infection and severe COVID-19’), Table 2, & Fig. 3 |
| Discussion | | |  |
| Key results | 18 | Summarise key results with reference to study objectives | Discussion, paragraphs 1-6 |
| Limitations | 19 | Discuss limitations of the study, taking into account sources of potential bias or imprecision. Discuss both direction and magnitude of any potential bias | Discussion, paragraphs 7-12 |
| Interpretation | 20 | Give a cautious overall interpretation of results considering objectives, limitations, multiplicity of analyses, results from similar studies, and other relevant evidence | Discussion, paragraph 13 |
| Generalisability | 21 | Discuss the generalisability (external validity) of the study results | Discussion, paragraph 10 |
| Other information | | |  |
| Funding | 22 | Give the source of funding and the role of the funders for the present study and, if applicable, for the original study on which the present article is based | Funding |

# **References**

1. Altarawneh HN, Chemaitelly H, Ayoub HH, Tang P, Hasan MR, Yassine HM, Al-Khatib HA, Smatti MK, Coyle P, Al-Kanaani Z *et al*: **Effects of Previous Infection and Vaccination on Symptomatic Omicron Infections**. *N Engl J Med* 2022, **387**(1):21-34.

2. Abu-Raddad LJ, Chemaitelly H, Ayoub HH, Al Kanaani Z, Al Khal A, Al Kuwari E, Butt AA, Coyle P, Jeremijenko A, Kaleeckal AH *et al*: **Characterizing the Qatar advanced-phase SARS-CoV-2 epidemic**. *Sci Rep* 2021, **11**(1):6233.

3. Abu-Raddad LJ, Chemaitelly H, Bertollini R, National Study Group for Covid Vaccination: **Effectiveness of mRNA-1273 and BNT162b2 Vaccines in Qatar**. *N Engl J Med* 2022, **386**(8):799-800.

4. Planning and Statistics Authority-State of Qatar: **Qatar Monthly Statistics. Available from:** [**https://www.psa.gov.qa/en/pages/default.aspx**](https://www.psa.gov.qa/en/pages/default.aspx)**. Accessed on: May 26, 2020**. 2020.

5. Chemaitelly H, Bertollini R, Abu-Raddad LJ, National Study Group for Covid Epidemiology: **Efficacy of Natural Immunity against SARS-CoV-2 Reinfection with the Beta Variant**. *N Engl J Med* 2021, **385**(27):2585-2586.

6. Chemaitelly H, Ayoub HH, AlMukdad S, Coyle P, Tang P, Yassine HM, Al-Khatib HA, Smatti MK, Hasan MR, Al-Kanaani Z *et al*: **Protection from previous natural infection compared with mRNA vaccination against SARS-CoV-2 infection and severe COVID-19 in Qatar: a retrospective cohort study**. *Lancet Microbe* 2022, **3**(12):e944-e955.

7. Mahmoud MA, Ayoub HH, Coyle P, Tang P, Hasan MR, Yassine HM, Al Thani AA, Al-Kanaani Z, Al-Kuwari E, Jeremijenko A *et al*: **SARS-CoV-2 infection and effects of age, sex, comorbidity, and vaccination among older individuals: A national cohort study**. *Influenza Other Respir Viruses* 2023, **17**(11):e13224.

8. Chemaitelly H, Ayoub HH, Tang P, Coyle PV, Yassine HM, Al Thani AA, Al-Khatib HA, Hasan MR, Al-Kanaani Z, Al-Kuwari E *et al*: **History of primary-series and booster vaccination and protection against Omicron reinfection**. *Sci Adv* 2023, **9**(40):eadh0761.

9. AlNuaimi AA, Chemaitelly H, Semaan S, AlMukdad S, Al-Kanaani Z, Kaleeckal AH, Latif AN, Al-Romaihi HE, Butt AA, Al-Thani MH *et al*: **All-cause and COVID-19 mortality in Qatar during the COVID-19 pandemic**. *BMJ Glob Health* 2023, **8**(5).

10. Vogels C, Fauver J, Grubaugh N: **Multiplexed RT-qPCR to screen for SARS-COV-2 B.1.1.7, B.1.351, and P.1 variants of concern V.3. dx.doi.org/10.17504/protocols.io.br9vm966**. 2021(June 6, 2021).

11. Abu-Raddad LJ, Chemaitelly H, Butt AA, National Study Group for Covid Vaccination: **Effectiveness of the BNT162b2 Covid-19 Vaccine against the B.1.1.7 and B.1.351 Variants**. *N Engl J Med* 2021, **385**(2):187-189.

12. Chemaitelly H, Yassine HM, Benslimane FM, Al Khatib HA, Tang P, Hasan MR, Malek JA, Coyle P, Ayoub HH, Al Kanaani Z *et al*: **mRNA-1273 COVID-19 vaccine effectiveness against the B.1.1.7 and B.1.351 variants and severe COVID-19 disease in Qatar**. *Nat Med* 2021, **27**(9):1614-1621.

13. **Qatar viral genome sequencing data. Data on randomly collected samples.** [**https://www.gisaid.org/phylodynamics/global/nextstrain/**](https://www.gisaid.org/phylodynamics/global/nextstrain/) [<https://www.gisaid.org/phylodynamics/global/nextstrain/>]

14. Benslimane FM, Al Khatib HA, Al-Jamal O, Albatesh D, Boughattas S, Ahmed AA, Bensaad M, Younuskunju S, Mohamoud YA, Al Badr M *et al*: **One Year of SARS-CoV-2: Genomic Characterization of COVID-19 Outbreak in Qatar**. *Front Cell Infect Microbiol* 2021, **11**:768883.

15. Hasan MR, Kalikiri MKR, Mirza F, Sundararaju S, Sharma A, Xaba T, Lorenz S, Chemaitelly H, El-Kahlout RA, Tsui KM *et al*: **Real-Time SARS-CoV-2 Genotyping by High-Throughput Multiplex PCR Reveals the Epidemiology of the Variants of Concern in Qatar**. *Int J Infect Dis* 2021, **112**:52-54.

16. Chemaitelly H, Tang P, Hasan MR, AlMukdad S, Yassine HM, Benslimane FM, Al Khatib HA, Coyle P, Ayoub HH, Al Kanaani Z *et al*: **Waning of BNT162b2 Vaccine Protection against SARS-CoV-2 Infection in Qatar**. *N Engl J Med* 2021, **385**(24):e83.

17. Saththasivam J, El-Malah SS, Gomez TA, Jabbar KA, Remanan R, Krishnankutty AK, Ogunbiyi O, Rasool K, Ashhab S, Rashkeev S *et al*: **COVID-19 (SARS-CoV-2) outbreak monitoring using wastewater-based epidemiology in Qatar**. *Sci Total Environ* 2021, **774**:145608.

18. El-Malah SS, Saththasivam J, Jabbar KA, K KA, Gomez TA, Ahmed AA, Mohamoud YA, Malek JA, Abu Raddad LJ, Abu Halaweh HA *et al*: **Application of human RNase P normalization for the realistic estimation of SARS-CoV-2 viral load in wastewater: A perspective from Qatar wastewater surveillance**. *Environ Technol Innov* 2022, **27**:102775.

19. Abu-Raddad LJ, Chemaitelly H, Ayoub HH, AlMukdad S, Yassine HM, Al-Khatib HA, Smatti MK, Tang P, Hasan MR, Coyle P *et al*: **Effect of mRNA Vaccine Boosters against SARS-CoV-2 Omicron Infection in Qatar**. *N Engl J Med* 2022, **386**(19):1804-1816.

20. Tang P, Hasan MR, Chemaitelly H, Yassine HM, Benslimane FM, Al Khatib HA, AlMukdad S, Coyle P, Ayoub HH, Al Kanaani Z *et al*: **BNT162b2 and mRNA-1273 COVID-19 vaccine effectiveness against the SARS-CoV-2 Delta variant in Qatar**. *Nat Med* 2021, **27**(12):2136-2143.

21. Altarawneh HN, Chemaitelly H, Hasan MR, Ayoub HH, Qassim S, AlMukdad S, Coyle P, Yassine HM, Al-Khatib HA, Benslimane FM *et al*: **Protection against the Omicron Variant from Previous SARS-CoV-2 Infection**. *N Engl J Med* 2022, **386**(13):1288-1290.

22. Chemaitelly H, Ayoub HH, AlMukdad S, Coyle P, Tang P, Yassine HM, Al-Khatib HA, Smatti MK, Hasan MR, Al-Kanaani Z *et al*: **Duration of mRNA vaccine protection against SARS-CoV-2 Omicron BA.1 and BA.2 subvariants in Qatar**. *Nat Commun* 2022, **13**(1):3082.

23. Qassim SH, Chemaitelly H, Ayoub HH, AlMukdad S, Tang P, Hasan MR, Yassine HM, Al-Khatib HA, Smatti MK, Abdul-Rahim HF *et al*: **Effects of BA.1/BA.2 subvariant, vaccination and prior infection on infectiousness of SARS-CoV-2 omicron infections**. *J Travel Med* 2022, **29**(6).

24. Altarawneh HN, Chemaitelly H, Ayoub HH, Hasan MR, Coyle P, Yassine HM, Al-Khatib HA, Smatti MK, Al-Kanaani Z, Al-Kuwari E *et al*: **Protective Effect of Previous SARS-CoV-2 Infection against Omicron BA.4 and BA.5 Subvariants**. *N Engl J Med* 2022, **387**(17):1620-1622.

25. Chemaitelly H, Tang P, Coyle P, Yassine HM, Al-Khatib HA, Smatti MK, Hasan MR, Ayoub HH, Altarawneh HN, Al-Kanaani Z *et al*: **Protection against Reinfection with the Omicron BA.2.75 Subvariant**. *N Engl J Med* 2023, **388**(7):665-667.

26. Chemaitelly H, Coyle P, Kacem MAB, Ayoub HH, Tang P, Hasan MR, Yassine HM, Thani AAA, Al-Kanaani Z, Al-Kuwari E *et al*: **Protection of natural infection against reinfection with SARS-CoV-2 JN.1 variant**. *medRxiv* 2024:2024.2002.2022.24303193.

27. Al-Thani MH, Farag E, Bertollini R, Al Romaihi HE, Abdeen S, Abdelkarim A, Daraan F, Elhaj Ismail AIH, Mostafa N, Sahl M *et al*: **SARS-CoV-2 Infection Is at Herd Immunity in the Majority Segment of the Population of Qatar**. *Open Forum Infect Dis* 2021, **8**(8):ofab221.

28. World Health Organization (WHO): **Living guidance for clinical management of COVID-19. Aavailable from:** [**https://www.who.int/publications/i/item/WHO-2019-nCoV-clinical-2021-2**](https://www.who.int/publications/i/item/WHO-2019-nCoV-clinical-2021-2)**. Accessed on: February 27, 2023.** 2021.

29. World Health Organization (WHO): **International Guidelines for Certification and Classification (Coding) of COVID-19 as Cause of Death. Available from:** [**https://www.who.int/publications/m/item/international-guidelines-for-certification-and-classification-(coding)-of-covid-19-as-cause-of-death**](https://www.who.int/publications/m/item/international-guidelines-for-certification-and-classification-(coding)-of-covid-19-as-cause-of-death)**. Accessed on: February 27, 2023.** 2020.
